# Evaluating the impact of integrated behavioral health intervention: Evidence from Rhode Island

**DOI:** 10.1097/MD.0000000000027066

**Published:** 2021-08-27

**Authors:** Bishnu Bahadur Thapa, M. Barton Laws, Omar Galárraga

**Affiliations:** Department of Health Services, Policy and Practice, School of Public Health, Brown University, Providence, RI.

**Keywords:** cost, integrated behavioral health, primary care, 2-part model, utilization

## Abstract

There has been a historic separation between systems that address behavioral health problems and the medical care system that addresses other health issues. Integration of the 2 has the potential to improve care.

The aim of this study was to evaluate the impact of Integrated Behavioral Health program on health care utilization and costs.

Claims data between 2015 and 2018 from Rhode Island's All Payers Claims Database representing 42,936 continuously enrolled unique patients.

Retrospective study based on propensity score–matched difference-in-differences framework.

Utilization (emergency department visits, office visits, and hospitalizations) and costs (total, inpatient, outpatient, professional, and pharmacy).

Integrated Behavioral Health intervention in Rhode Island was associated with reduction in healthcare utilization. Emergency department visits reduced by 6.4 per 1000 people per month and office visits reduced by 29.8 per 1000 people per month, corresponding to a reduction of 7% and 6%, respectively. No statistically significant association was observed between the intervention and hospitalizations. The evidence was mixed for cost outcomes, with negative association recorded between the intervention and the likelihood of incurring non-zero cost but no significant association was observed between the intervention and the level of costs. This relationship held true for most of the cost measures considered.

Integrated Behavioral Health intervention in Rhode Island was associated with significant reductions in emergency department visits and office visits, with no effects on hospitalizations. In terms of the cost outcomes, we found evidence that the intervention negatively affected the likelihood of incurring any non-zero costs but did not affect the level of costs.

## Abstract 2

Supplemental Digital Content is available in the text

## 1 Introduction

### 1.1 Background

Behavioral health (BH) conditions include issues related to “the promotion of mental health, resilience and wellbeing; the treatment of mental and substance use disorders; and the support of those who experience and/or are in recovery from these conditions, along with their families and communities.”^[1,2]^ BH conditions are highly prevalent and were the leading cause of disease burden in the United States in 2015.^[3]^ In 2017, BH-linked comorbidities accounted for nearly $406 billion in additional health care cost.^[4]^ Despite their widespread prevalence, these conditions are neither sufficiently diagnosed nor receive timely treatment.^[5]^

At a broad level, the magnitude of the BH challenge in the United States is linked to the historic separation that exists between systems that address BH conditions and the medical care system which addresses other health issues. These systems differ in organization and financing, and are often represented by separate institutions and different professions. Such separation has meant that BH services are often provided separately from primary care.^[5]^ Evidence suggests that effective medical management, social support, and patient experience are greatly enhanced when behavioral health and, primary care, and medical services are integrated.^[6]^

Integrated behavioral health is a care delivery model that combines or coordinates care for physical, mental, and substance use disorders, usually to address problems identified during primary care visits.^[7]^ Despite the growing number of interventions and initiatives to integrate BH into primary care, rigorous evidence on the impacts of integrated behavioral health care delivery models within the context of primary care remains limited.^[8]^ This article addresses the evidence gap on the effects of integrating behavioral health into primary care on health care utilization and cost. Using propensity score-matched difference-in-differences (DiD) research design, this article examined the effects of an integrated behavioral health program in Rhode Island on health care utilization and cost.

### 1.2 The intervention

Integrated Behavioral Health (IBH) program in Rhode Island was a practice-level intervention rolled out across 11 primary care practices in the state. The intervention entailed a number of inter-related components including: the universal screening of patients for depression, anxiety, and substance use disorders; hiring BH providers (eg, clinical social workers or psychologists) onsite; tracking and treating (eg, through counseling and therapy) patients who are screened positive at baseline (ie, those who suffered from relatively higher levels of depression, anxiety or substance use disorders s when first screened); making referrals to specialty care (eg, psychiatry care) as needed; and care coordination across different types of care. Participating practices used the following tools to screen patients: Patient Health Questionnaire-2/Patient Health Questionnaire-9, General Anxiety Disorder-7 and CAGE Adopted to Include Drugs. Based on their screening scores, patients were referred to a BH provider onsite. As a part of the IBH program, participating practices also received financial payments towards infrastructure support and a performance incentive for meeting pre-identified screening thresholds for depression, anxiety and substance disorder use.^[9]^

To participate in the IBH program, the practices needed to be a Patient Centered Medical Home and had to have achieved National Committee for Quality Assurance (NCQA) Level 2 recognition. The participating practices also had access to and participated in on-site IBH trainings and webinars, with the goal of helping the practices implement an IBH program that provided holistic patient-centered primary care services.

The IBH program was rolled out in 2 waves across the 11 primary care practices. As a part of the first wave, 6 primary care practices received the intervention between January 2016 and December 2017. In the second wave, 5 practices received the intervention between November 2016 and October 2018. The intervention lasted for 2 years in both the waves. For the purposes of this article, we focused on the first wave of the intervention.

The IBH program was implemented by the Care Transformation Collaborative of Rhode Island in 2016. Since its inception in 2008, Care Transformation Collaborative of Rhode Island has embarked on a number of initiatives, including Patient-Centered Medical Homes and Community Health Teams (CHTs). The IBH program was one of such initiatives aimed at improving primary care in Rhode Island.

## 2 Methods

### 2.1 Data and sample

We used Rhode Island's All Payers Claims Database, which includes medical and pharmacy claims from Medicare, Medicaid and commercial payers in the state. We used data between January 2015 and December 2018. Our analytical sample comprised of 42,936 adults who were 18 years and older and were continuously enrolled between 2015 and 2018.

### 2.2 Intervention group definition

The intervention group consisted of people who received healthcare from the primary care practices that participated in the IBH program. This included all the individuals who received services from the 6 practices that made the first wave of the IBH program. Comparison group consisted of a matched set of individuals that resembled the intervention group in terms of its baseline characteristics. The 12 months’ window preceding the intervention start date was considered as the pre-period (ie, the baseline).

### 2.3 Outcome measures

We looked at 2 sets of outcomes: utilization and cost. Utilization was measured by office visits, emergency department (ED) visits, and inpatient visits or hospitalizations. For cost outcomes, we looked at 5 measures: total cost, inpatient cost, outpatient cost, pharmacy cost, and professional cost. The cost measures were based on the 2017 Health Care Cost and Utilization Report.^[10]^ All outcome measures were analyzed at the person-month level.

### 2.4 Empirical approach

We employed the propensity score matching (PSM) technique to identify a comparison group that was similar to the intervention group. The DiD framework was then used to identify the changes in outcomes associated with the intervention.

In the absence of an experimental data, PSM can be used to make observational data mimic, to some extent, data from a randomized control trial.^[11]^ This is achieved mainly by matching on observable characteristics. Unlike a randomized control trial, however, PSM cannot match on unobservable characteristics. In our data, selection into receiving the IBH intervention was non-random. By design, the intervention was offered only to those practices that chose to participate (and by extension, patients who visited those practices). We used the PSM technique to create a matched comparison group.

To implement the PSM technique, we estimated a propensity score based on a bivariate probit model. The propensity score was then used to create a matched comparison group with observable baseline characteristics similar to the intervention group. We matched at the individual level using one-to-many matching with replacement.^[12]^ Our matching variables included *age, sex, Medicare status, Medicare status category, dual status, Medicaid status category, and Elixhauser Comorbidity Index*. The variables were selected because they were identified, based on the existing literature on health services research literature, as potential confounders that bias intervention effect size estimates. We examined covariate balance and analyzed graphically the region of common support to ensure that the intervention group (N = 12,298) and the comparison group (N = 30,638) at baseline looked similar in terms of their baseline characteristics.

Both utilization and cost outcomes were modeled using generalized estimating equations.^[13,14]^ For cost outcomes, we used the 2-part model, in which the first part models the probability of non-zero costs and the second part models the level of costs for observations with non-zero costs. This approach of modeling cost has been described and used widely in the existing literature.^[15–17]^ We assumed binomial distribution with log-link to model the first part.^[18]^ The second part was modeled assuming gamma distribution with log link. Modeling of the utilization outcome assumed negative binomial distribution with log-link.^[19]^

The following framework was used to estimate the effects of the intervention on outcomes:


(1) $$Y_{im}=\alpha +\beta (IBH\;Status_{i})+\gamma (Post_{m})+\delta (IBH\;Status_{i}*Post_{m})+\sigma X+v_{m}+\varepsilon_{im}$$

where *Y*_*im*_ represents an outcome (utilization or cost) for person *i* in month *m*. For both utilization and cost models, we included an indicator variable to denote whether someone received services from an IBH practice (*IBH Status*); an indicator variable for the post period (*= 1 if intervention period, ie, between January 2016 and December 2017*); and an interaction term between IBH Status and Post *(IBH Status ∗ Post).* The coefficient on the interaction term (*δ*) is the coefficient of our interest and represents the DiD estimator. Vector ***X*** includes *age, sex, Medicaid coverage, Medicaid eligibility basis, Medicare coverage, dual eligibility, zip code-level poverty rates, and Elixhauser Comorbidity Index in the baseline period*.

Our models are based on analyses at the person-month level. The models accounted for one-to-many matching, with replacement, by using frequency weights.^[20]^ We controlled for time trends using monthly fixed effects (*v*_*m*_), and used robust standard errors and individual-level clustering to account for the repeated nature of the data. The DiD estimates are presented as the mean marginal effects, with the exception that the DiD estimates for part one of the 2-part model for cost measures are presented as the odds ratios (ORs). This was done because ORs are relatively easier to estimate and interpret.^[19,21,22]^

As a part of our sensitivity analyses, we did 2 things. First, to test the parallel trends assumption, we graphically analyzed the linear trends of the outcome measures for the intervention group and the comparison group. Second, we carried out an analysis to check whether there was any kind of “maintenance effect” (that is, we examined whether the effect of IBH became stronger over time). To do this, we re-defined the post period to include both the actual intervention period as well as the additional time until the end of 2018. Results of the sensitivity analyses are included in supplementary digital content (SDC, http://links.lww.com/MD2/A349), in parts B and C.

All analyses were conducted using Stata version 16 (StataCorp). The study was approved by the Institutional Review Board of Brown University.

## 3 Results

### 3.1 Covariate balance

As shown in Table 1, baseline characteristics between the intervention group and the matched comparison group were very similar for nearly all the characteristics considered. Of the study population, 58% was Medicaid-eligible, 23% was Medicare-eligible, and 10% was dual eligible. Average age was just over 45 years and females comprised of nearly two-thirds of the study population. Zip code-level poverty rate, the only baseline characteristic with a statistically significant *P* value, was higher for the comparison group than the intervention group.

**Table 1:** Characteristics at baseline (2015).

Variable	Comparison	Intervention	*P*
Age, y	45.57	45.67	.63
Female (%)	0.678	0.674	.46
Dual status (%)	0.098	0.101	.47
Elixhauser comorbidity index	1.206	1.223	.33
Medicaid (%)	0.578	0.582	.57
Medicaid category (%)			
Blind/disabled	0.126	0.123	.53
Parents/caretakers	0.182	0.179	.56
Children	0.011	0.012	.48
Expansion adults	0.194	0.194	.95
Non-Medicaid	0.432	0.430	.66
Medicare (%)	0.223	0.228	.31
Medicare category (%)			
Aged without ESRD	0.128	0.129	.89
Disabled without ESRD	0.094	0.098	.26
Non-Medicare	0.777	0.772	.31
Poverty rate	16.232	14.173	.00
N	30,638	12,298	

The *P* value reflects the significance of the difference in means between the intervention and the comparison group. ESRD = end-stage renal disease. Elixhauser Comorbidity Index is a summary measure for the # of comorbidities that a patient has. Dual status means patient has both Medicaid and Medicare coverage. Only the major categories are shown under Medicaid/Medicare categories.

### 3.2 Association of IBH with utilization and cost

IBH intervention was associated with a significant reduction in ED visits and office visits but with no change in hospitalizations (Table 2). ED visits reduced by an average of 6.4 per 1000 people per month (*P* = .004), representing a percent reduction of 7%. Office visits reduced by an average of 30 visits per 1000 people per month (*P* < .001) and represented a percent reduction of 6%. The coefficient on hospitalizations was negative but not statistically significant.

**Table 2:** Association between IBH intervention and utilization.

	Comparison		Intervention		
Outcome	Pre	Post	Pre	Post	Difference-in-differences
ED visits	93	88	95	84	−6.4^∗∗^ (−10.9 to −1.98)
Office visits	471	490	505	494	−29.8^∗∗∗^ (−40 to −19.7)
Hospitalizations	22	21	19	18	−0.3 (−2 to 1.53)

Table shows # of ED visits, office visits and hospitalizations per 1000 people per month before (pre) and after (post) the intervention. Difference-in-differences estimates (and their 95% confidence intervals) are shown at the right most column. IBH = integrated behavioral health. The value of N, representing person months for the study window was 1,510,786.

∗∗ *P* < 0.01.

∗∗∗ *P* < 0.001.

We found a statistically significant association of the IBH intervention with the probability of having non-zero costs. For observations with non-zero costs, however, we did not find any significant associations of the intervention with the level of costs. This pattern held true for nearly all measures of cost. As shown in Table 3, IBH intervention was associated with significantly lower odds of total cost, outpatient cost, professional cost, and pharmacy cost. The odds of having non-zero costs were 0.92 (*P* < .001), 0.94 (*P* < .001), 0.93 (*P* < 0.001), and 0.97 (*P* = .03) for total cost, outpatient cost, professional cost, and pharmacy cost, respectively. There was no significant association between the intervention and the odds of having non-zero inpatient costs. Conditional on having non-zero costs, however, the IBH intervention had no significant association with the level of costs.

**Table 3:** Association between IBH intervention and cost.

	Comparison		Intervention		
Outcome	*Pre*	*Post*	*Pre*	*Post*	Difference-in-differences
Total cost of care					
Model: Part I	0.685	0.695	0.709	0.704	0.922^∗∗∗^ (0.891 to 0.955)
Model: Part II	1320.7	1464.3	1123.5	1228.6	−38.556 (−135.2 to 58.1)
Inpatient					
Model: Part I	0.019	0.022	0.015	0.016	0.953 (0.875 to 1.037)
Model: Part II	24299.4	18883.2	26845.7	20517.2	−912.1 (−4546.6 to 2722.3)
Outpatient					
Model: Part I	0.210	0.200	0.222	0.202	0.936^∗∗∗^ (0.908 to 0.965)
Model: Part II	682.3	650.1	651.0	625.2	6.260 (−37.3 to 49.8)
Professional					
Model: Part I	0.508	0.494	0.519	0.488	0.926^∗∗∗^ (0.900 to 0.952)
Model: Part II	493.6	477.3	444.7	422.8	−5.7 (−20.8 to 9.5)
Pharmacy					
Model: Part I	0.504	0.567	0.514	0.569	0.966^∗∗^(0.936 to 0.996)
Model: Part II	260.6	350.0	195.4	264.9	−19.9 (−48.7 to 8.9)

Part I models the probability of observing non-zero costs while part II models the level of costs for those with non-zero costs. Difference-in-differences estimates for part I represent odds ratios while for part II represent mean marginal monthly cost per person. IBH = integrated behavioral health. The size of N, which represents the # of person-months during the study window, for part I is 1,510,786 and for part II, averages 655,059 across the 5 cost measures considered. IBH = Integrated Behavioral Health.

∗∗ *P* < 0.01.

∗∗∗ *P* < 0.001.

Results of the sensitivity analysis were consistent with the main results (see SDC parts B and C, http://links.lww.com/MD2/A349). As shown in SDC, http://links.lww.com/MD2/A349 part C table A1, http://links.lww.com/MD2/A349, the significant association observed between the IBH intervention and the utilization and cost measures was sustained even a year after the end of the intervention. ED visits reduced by 7.6 per 1000 people per month (*P* < .01), whereas office visits reduced by 31.6 per 1000 people per month (*P* < .001). No significant association was observed with respect to hospitalizations. In terms of the cost measures, IBH was associated with a decrease in the odds of incurring non-zero costs, with the ORs ranging between 0.86 and 0.96. Inpatient cost was the only exception. For a given level of non-zero cost, no significant association was observed between IBH intervention and the level of cost. This relationship held true across all cost categories.

## 4 Discussion

Relative to the comparison group, the IBH intervention group saw reduction in monthly office and ED visits. Mean reduction in office visits was about 29.8 per 1000 people per month and the mean reduction in ED visits was about 6.3 per 1000 people per month. There was no association between the IBH intervention and hospitalizations.

There could be a number of reasons for the observed reduction in ED and office visits. First, it could be that the IBH intervention generally discouraged ED visits for unnecessary care. Second, the IBH intervention may have enabled the primary care practices to increase both the overall volume of their services as well as the amount of effort in delivering those services. The change in both volume and the quality of services offered at the practices may have reduced the need for ED visits as well as the frequency of office visits.

The intervention showed mixed associations with respect to cost measures. The intervention group had lower odds of incurring non-zero costs relative to the comparison group. This was true for all cost measures, except inpatient costs. The magnitude of the decrease in odds ranged between 3% and 8%. Conditional on incurring non-zero costs, however, the intervention had no association with the level of cost for any of the cost measures. The results on costs suggest that the IBH intervention reduced costs on the extensive margin but had no effects on the intensive margin.^[23]^

Existing studies based on similar interventions suggest generally positive association with utilization measures but mixed association with cost measures. Findings from our study are consistent with studies based on similar interventions. For example, a randomized study based on Kaiser Sacramento showed decreased ED use for patients who received an IBH-like intervention. However, unlike ours, that study showed decrease in hospitalizations as well as level of costs for these patients. A shortcoming of the study, however, was that it was based on a small sample size (n = 654).^[24]^ A comparative effectiveness-based evaluation of the Primary and Behavioral Health Care Integration Small Grants Program by the RAND Corporation showed increased access to integrated care, but the evidence on the improvement of the health indicators was mixed.^[25]^ Another study based on Intermountain Healthcare System's Mental Health Integration program showed that patients who received integrated care saw a reduction in healthcare utilization. This was manifest in lower ED visits, hospitalizations, and physician-office visits. Furthermore, the integrated care was also associated with reduced program costs, an aspect that is beyond the scope of our study.^[26]^ When Cherokee Health Systems in Tennessee introduced BH services into its care delivery system by co-locating BH providers in its primary care settings, it saw considerable reductions in ED visits, hospitalizations, specialty care, and total cost of care.^[27]^

Our study has limitations. First, the IBH program was a practice-level intervention but our analysis is at the person-level. This means that it is possible for someone without any BH issues to visit an IBH practice and receive care that is unrelated to the IBH intervention itself. Yet, the person can be flagged as an IBH person in our sample simply because he/she visited an IBH practice. If/when this occurred, however, the potential bias would be toward the null. Second, the intervention had a number of inter-related components to it. Although some components were likely more important than other components, we cannot disentangle the effects of the different components of the intervention. The results presented are the combined effects of the components that make up the intervention. As such, the exact results are a lower bound of the most active components that may have been “watered down” by the inability to separate. Third, in implementing the DiD strategy, we pool all the pre periods into one period, and pool all the post periods into one period. While this is an accepted practice in the literature and allows us to compare pre and post outcomes means between the intervention and the comparison group, we lose some granularity in the process. Fourth, in looking at the utilization outcomes, we are unable to disentangle BH-linked utilization from overall utilization. For example, we cannot say the share of office visits that were directly attributable to BH conditions.

Our study has important implications for health policy and practice. We showed that there was reduction in ED and office visits associated with the IBH intervention. This suggests the potential role of IBH interventions in reducing unnecessary ED and office visits, thereby saving scarce resources. Although we mostly saw no effects of the intervention on the level of costs, our finding that the intervention was associated with reduction in the odds of incurring non-zero costs bodes well for the possible role of IBH interventions in reducing overall costs. Future research in the area should try to quantify and contextualize the effects of IBH-like interventions on necessary care versus care that is deemed not as necessary.

## Acknowledgments

The authors thank Deepak Adhikari, MS and Xinqi Li, PhD, for their analytical insights. The authors also thank Debra Hurwitz, MBA and Pano Yeracaris, MD, for their insights on the IBH program.

## Author contributions

**Conceptualization:** Michael Barton Laws, Omar Galarraga.

**Data curation:** Bishnu Bahadur Thapa.

**Formal analysis:** Bishnu Bahadur Thapa, Omar Galarraga.

**Funding acquisition:** Omar Galarraga.

**Investigation:** Bishnu Bahadur Thapa, Michael Barton Laws, Omar Galarraga.

**Methodology:** Bishnu Bahadur Thapa, Michael Barton Laws, Omar Galarraga.

**Project administration:** Omar Galarraga.

**Resources:** Michael Barton Laws, Omar Galarraga.

**Supervision:** Michael Barton Laws, Omar Galarraga.

**Writing – original draft:** Bishnu Bahadur Thapa, Michael Barton Laws, Omar Galarraga.

**Writing – review & editing:** Bishnu Bahadur Thapa, Michael Barton Laws, Omar Galarraga.
